# Wavelength-Controlled Photodetector Based on Single CdSSe Nanobelt

**DOI:** 10.1186/s11671-018-2590-6

**Published:** 2018-06-07

**Authors:** Xinmin Li, Qiuhong Tan, Xiaobo Feng, Qianjin Wang, Yingkai Liu

**Affiliations:** 1 Institute of Physics and Electronic Information, Yunnan Normal University, Kunming, 650500 People’s Republic of China; 2 Yunnan Key Laboratory of Opto-electronic Information Technology, Kunming, 650500 People’s Republic of China; 3 Key Laboratory of Yunnan Higher Education Institutes for Optoelectric Information Technology, Kunming, 650500 People’s Republic of China

**Keywords:** CdSSe nanobelts, Optical properties, Cathodoluminescence, Photodetector

## Abstract

CdSSe nanobelts (NBs) are synthesized by thermal evaporation and then characterized by scanning electron microscopy (SEM), X-ray diffraction (XRD), transmission electron microscopy (TEM), high-resolution electron microscopy (HRTEM), X-ray photoelectron spectroscopy (XPS), photoluminescence (PL), and cathodoluminescence (CL). It is found that the CdSSe NBs have a good morphology and microstructure without defects. CL is sensitive to the defects of CdSSe NBs; thus, we can select single nanobelt with homogeneous CL emission to prepare a detector. Based on it, the photodetector of single CdSSe NB was developed and its photoelectric properties were investigated in detail. It is found that under illumination of white light and at the bias voltage of 1 V, the photocurrent of a single CdSSe nanobelt device is 1.60 × 10^−7^ A, the dark current is 1.96 × 10^−10^ A, and the ratio of light current to dark one is 816. In addition, the CdSSe nanobelt detector has high photoelectric performance with spectral responsivity of 10.4 AW^−1^ and external quantum efficiency (EQE) of 19.1%. Its rise/decay time is about 1.62/4.70 ms. This work offers a novel strategy for design wavelength-controlled photodetectors by adjusting their compositions.

## Background

Recently, semiconductor nanomaterials have been widely studied as optoelectronic devices, such as light emitting diodes [1, 2], photovoltaic devices [3], solar cell [4, 5], electrocatalytic H_2_ generation [6, 7], and photodetectors [8–10]. CdS and CdSe are II–VI semiconductor materials with the bandgap at room temperature of 2.42 and 1.74 eV, respectively. They are considered to be the best materials for the fabrication of photodetectors due to their bandgap corresponding to the absorption wavelength in the visible light region [11, 12].

One-dimensional nanostructures such as nanowires [13], nanobelts [14], and nanotubes [15] have been used in sensors and photodetectors due to their high surface-to-volume ratios, physical properties, and chemical properties [16]. Among them, some nanostructures such as ZnO [17], CdS [18], CdSe [19], MoS_2_ [20], Zn_*x*_Cd_1 − *x*_Se [21], CdS_1 − *x*_Se_*x*_ [22], and Zn_*x*_Cd_1 − *x*_S [23] have been used in fabrication photodetectors. Pan et al. reported that the photodetector based on CdS_0.49_Se_0.51_/CdS_0.91_Se_0.09_ heterostructure has a good performance [24]. However, how to develop a high response and selectivity detector with excellent performance is still a challenge.

In this work, the CdSSe nanobelts (NBs) were synthesized by thermal evaporation. We undertake the fabrication and characterization of single CdSSe device. After that, the photoelectric properties of single CdSSe NB device were systematically investigated. Based on it, we carried out the cathodoluminescence (CL) of CdSSe NB at room temperature and low temperature and found that CL is sensitive to the defects of CdSSe NBs. Therefore, we choose nanobelts with perfect microstructures to design devices by CL so that they can achieve our desired properties.

## Methods

### Preparation of CdSSe Nanobelts

Single-crystal CdSSe NBs were prepared by thermal evaporation. For synthesis of CdSSe NBs, the mixture of pure CdS powders (99.99 wt%) and CdSe powders (9.99 wt%) premixed in the weight ratio of 1:1 was put into a ceramic boat. The ceramic boat was placed in the middle of the quartz tube. A silicon substrate coated with about 10-nm Au film was placed into the tube; the distance of silicon substrate and ceramic boat was about 5–7 cm. The furnace was heated to 820 °C and was then maintained for 2 h. Finally, the furnace naturally cooled to room temperature. The nanobelts with different compositions were deposited on the different position of the Si substrate. In the whole experiment, Ar gas was flown at 20 sccm, and the pressure inside the tube was kept at 112 Torr.

### Material Characterization

The morphology, structure, and composition of the nanobelts were characterized by scanning electron microscopy (SEM), X-ray diffraction (XRD), transmission electron microscopy (TEM), high-resolution electron microscopy (HRTEM), and X-ray photoelectron spectroscopy (XPS). The PL spectra were measured under 532-nm laser excitation. CL spectra of CdSSe NBs were measured at room temperature and low temperature by a cathodoluminescence (CL) (Gatan monocle CL4) system installed on the scanning electron microscopy (Quanta FEG 250).

### Fabrication of Nanobelt Device

**Fig. 1:**
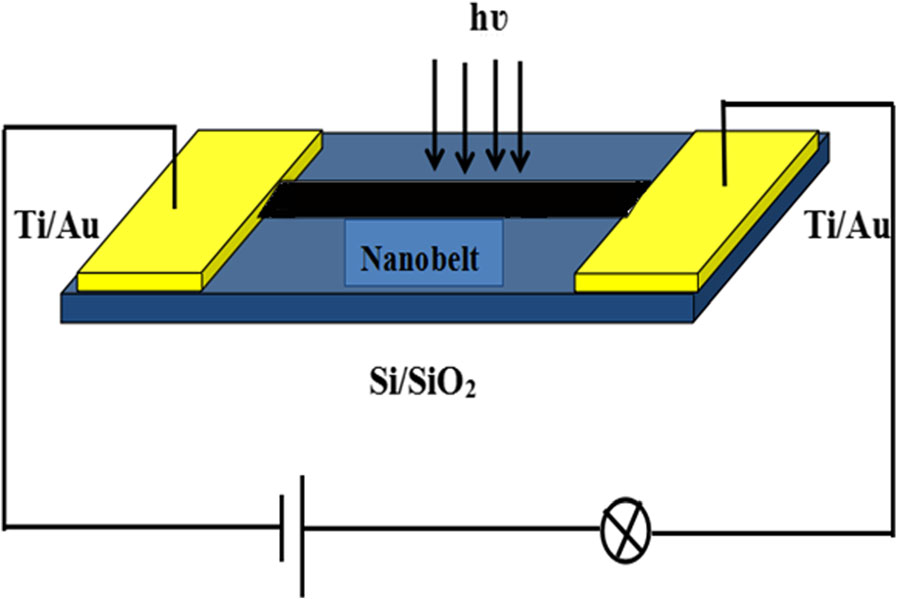
A schematic diagram of the detector configuration

Ti/Au electrodes were deposited on the two ends of a single nanobelt dispersed on Si substrate with a 500-nm-thick SiO_2_ layer, and then, the device was attained. The detailed fabrication process of the devices is referred in the literature [25]. The uncovered part of the nanobelts was exposed to the incident light. Figure 1 is the schematic diagram of the device test.

### Photoelectric Characterization

The measurement on the photoelectric performance of nanobelts was carried out by Keithley 4200 semiconductor system and the monochromatic spectrometer. The photocurrent of the device was measured by changing the incident light vertically irradiated on the device, and *I*–*V* curves were performed by a two-probe measurement.

## Results and Discussion

**Fig. 2:**
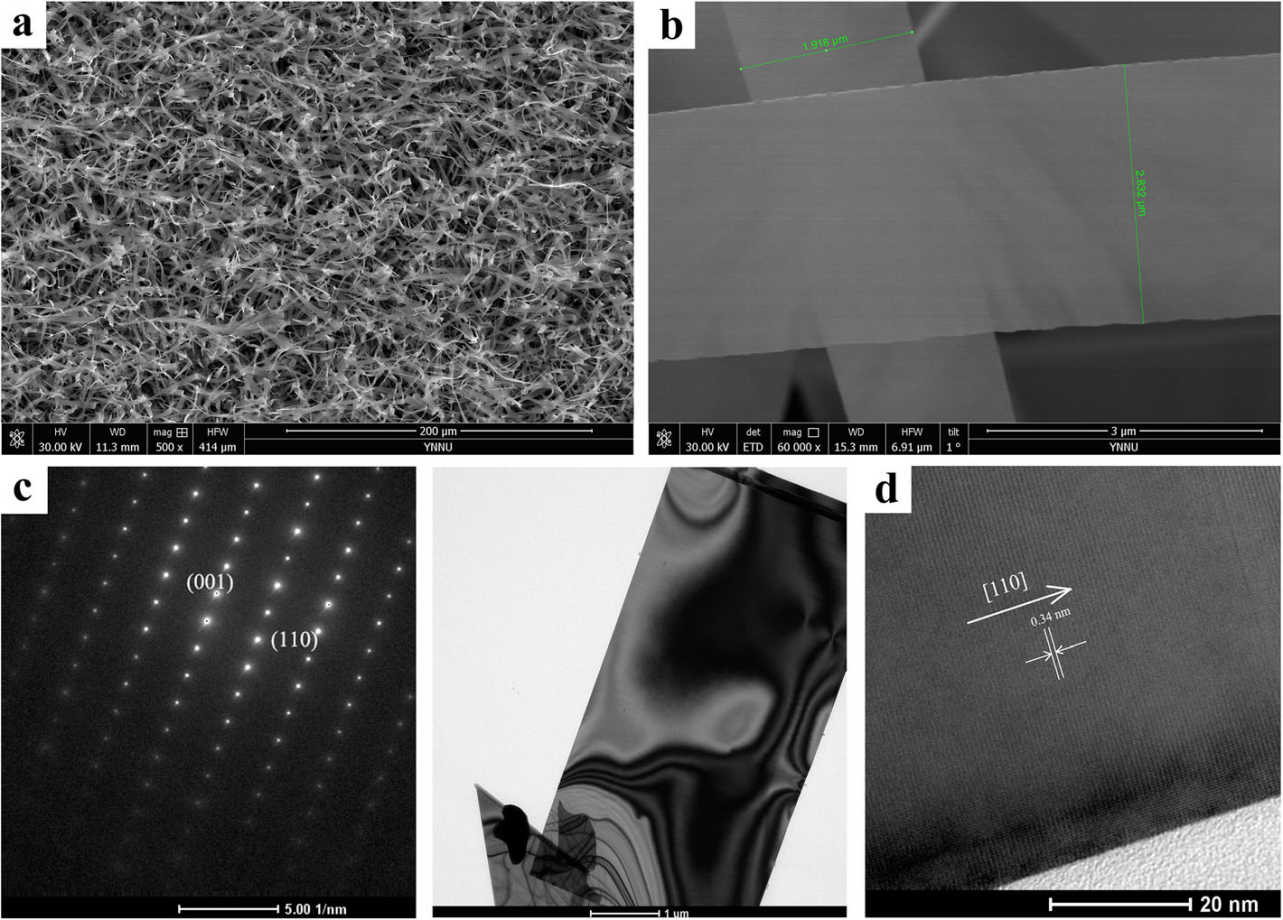
The morphology images of CdSSe NBs. **a** SEM at low magnification. **b** SEM at high magnification. **c** SAD, inset: its TEM. **d** HRTEM

Figure 2a shows a SEM image of the as-prepared CdSSe NBs. It is found that the CdSSe NBs have good morphology and uniform width and lengths up to hundreds of micrometers. Figure 2b is a high magnification SEM image of the CdSSe nanobelts. It is observed that the nanobelt is thin and uniform with 2.632 μm in width. Figure 2c and its inset present the TEM bright-field image and selected-area diffraction (SAD) pattern of a single nanobelt with a width of 2.94 μm and thickness of less than 50 nm. The SAD pattern confirms the single-crystal quality, and it can be indexed to a hexagonal structure with lattice parameters *a* = 4.177 Å and *c* = 6.776 Å. The corresponding HRTEM image is displayed in Fig. 2d, and the lattice spacing between adjacent plane is 0.34 nm, corresponding to the (110) crystal plane. Accordingly, its growth direction is along [110].

**Fig. 3:**
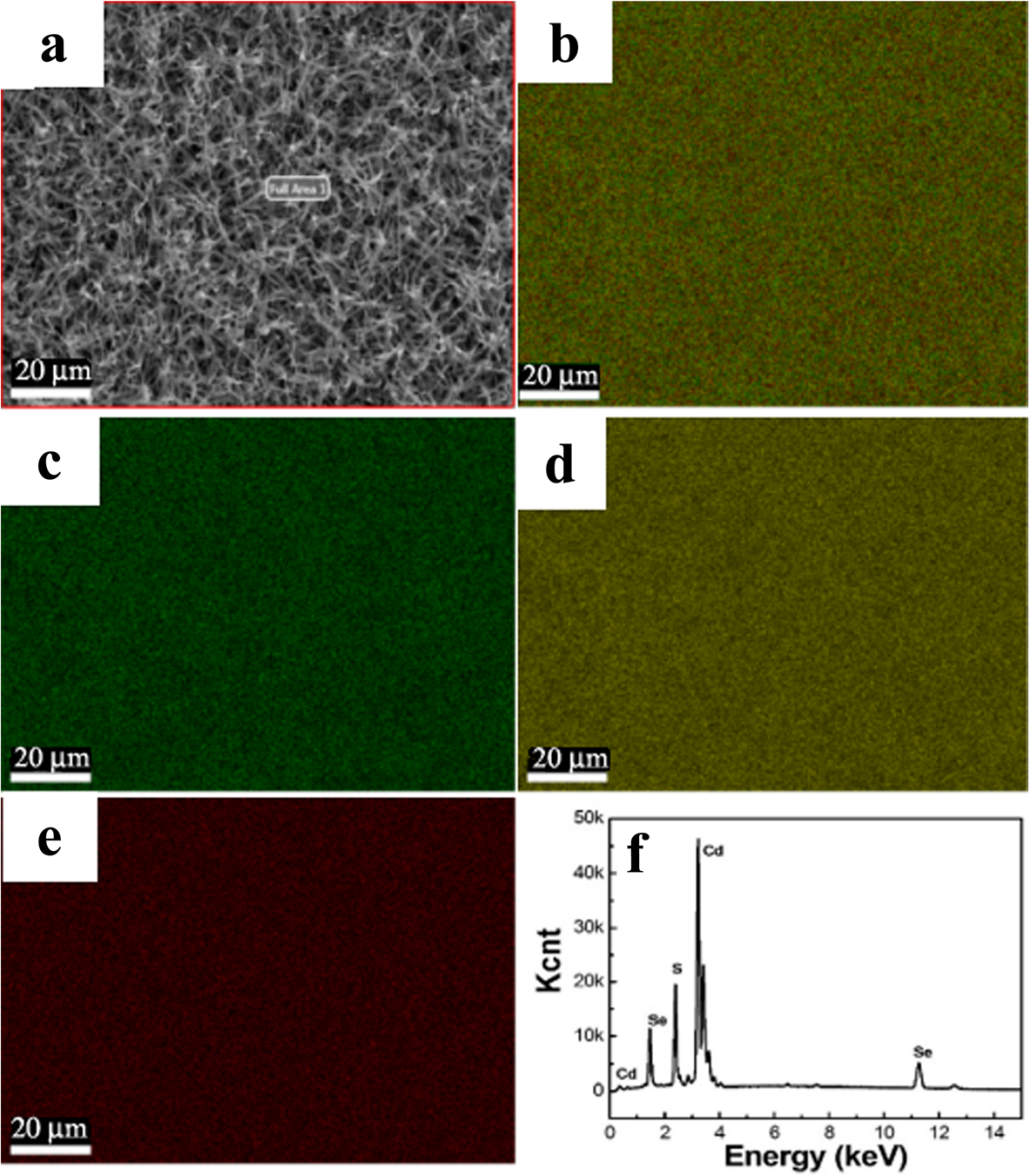
SEM image and elemental mappings of CdSSe NBs. **a** SEM. **b–e** Cd, S, and Se mappings, respectively. **f** EDX

EDX and mapping of the CdSSe nanobelts are shown in Fig. 3. SEM image of a sample at low multiples is displayed in Fig. 3a. It is observed that the entire region is covered with nanobelts. Figure 3b is the total distributions of Cd, S, and Se. The mappings of Cd, S, and Se elements are depicted in Fig. 3c–e, respectively. It revealed that Cd, S, and Se are uniformly distributed in the whole nanobelts. EDX spectrum collected from the same nanobelts is presented in Fig. 3f, indicating that the nanobelt is composed of Cd, S, and Se elements.

**Fig. 4:**
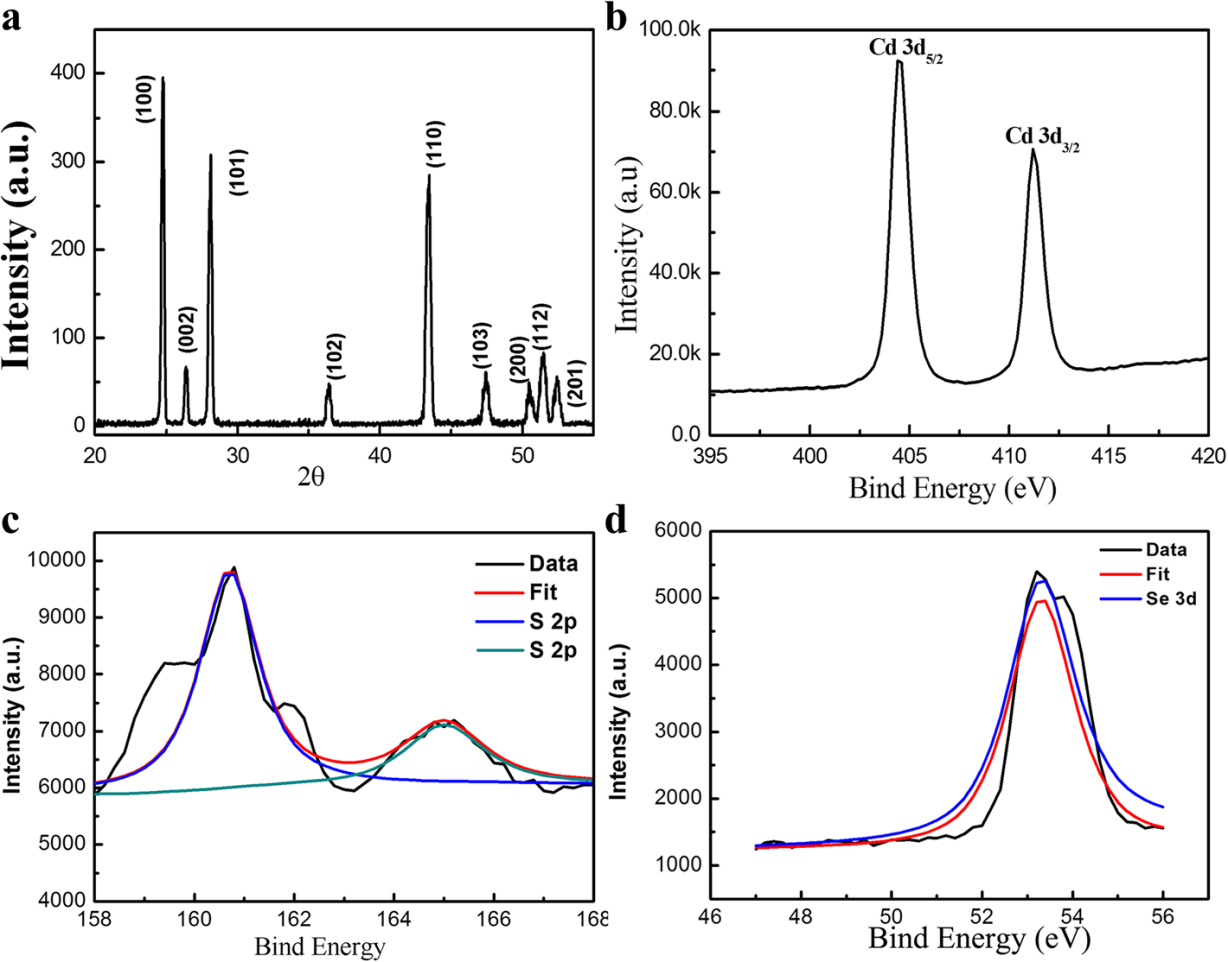
XRD patterns and XPS spectra of the CdSSe NBs. **a** XRD. **b** XPS spectrum of superposed Cd(3d). **c** High-resolution XPS spectrum for S(2p). **d** High-resolution XPS spectrum for Se(3d)

XRD and XPS pattern of CdSSe NBs are presented in Fig. 4. All diffraction peaks can be indexed to a hexagonal structure of CdS_0.76_Se_0.24_ with lattice parameters *a* = 4.177 Å and *c* = 6.776 Å, which is consistent with the standard card (JCPDS no. 49-1459). The positions of the diffraction peaks (2*θ* = 24.72°, 26.35°, 28.13°, 36.42°, 43.47°, 47.5°, 50.4°, 51.4°, and 52.4°) matched with the crystal plane (100), (002), (101), (102), (110), (103), (200), (112), and (201), respectively. No other impurities are detected. The sharp and narrow diffraction peaks revealed that the obtained CdSSe nanobelts have good crystallinity. Figure 4b shows the binding energies of Cd3d_5/2_ and Cd3d_3/2_ for the CdSSe NBs at 404.8 and 411.7 eV, respectively, which are close to the values reported in the previous work [26]. The separation distance between two peaks is 6.9 eV, indicating that Cd atoms are in the complete CdS phase [27]. The deconvolution of the S(2p) peak shows two Gaussian peaks, centered at 160.7 and 165.1 eV in Fig. 4c. The valence electron spectra of Se(3d) is depicted in Fig. 4d, in which only one peak located at 53.5 eV was observed. Therefore, the XPS results confirm that the nanobelts are composed of Cd, S, and Se elements.

**Fig. 5:**
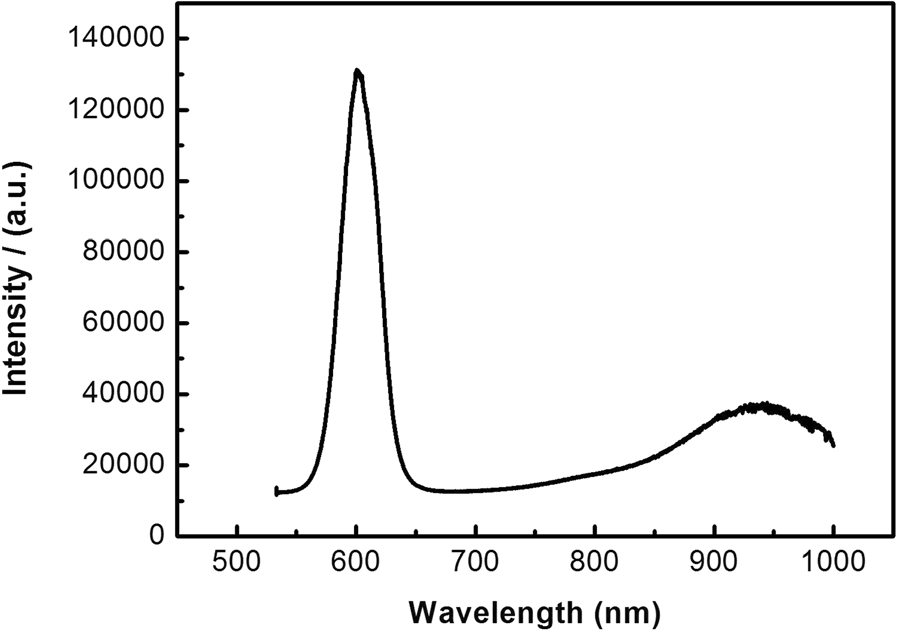
The PL emission spectra of CdSSe NBs

Figure 5 is the photoluminescence spectrum of CdSSe nanobelts; there are two peaks in the range of 500–1000 nm. One is centered at 603 nm originated from the near-band-edge (NBE) emission of the CdSSe nanobelts. The other centered at ~ 950 nm may be related to deep-level emission, which is observed in In_2_Se_3_ and Ga_2_Se_3_ [28, 29].

**Fig. 6:**
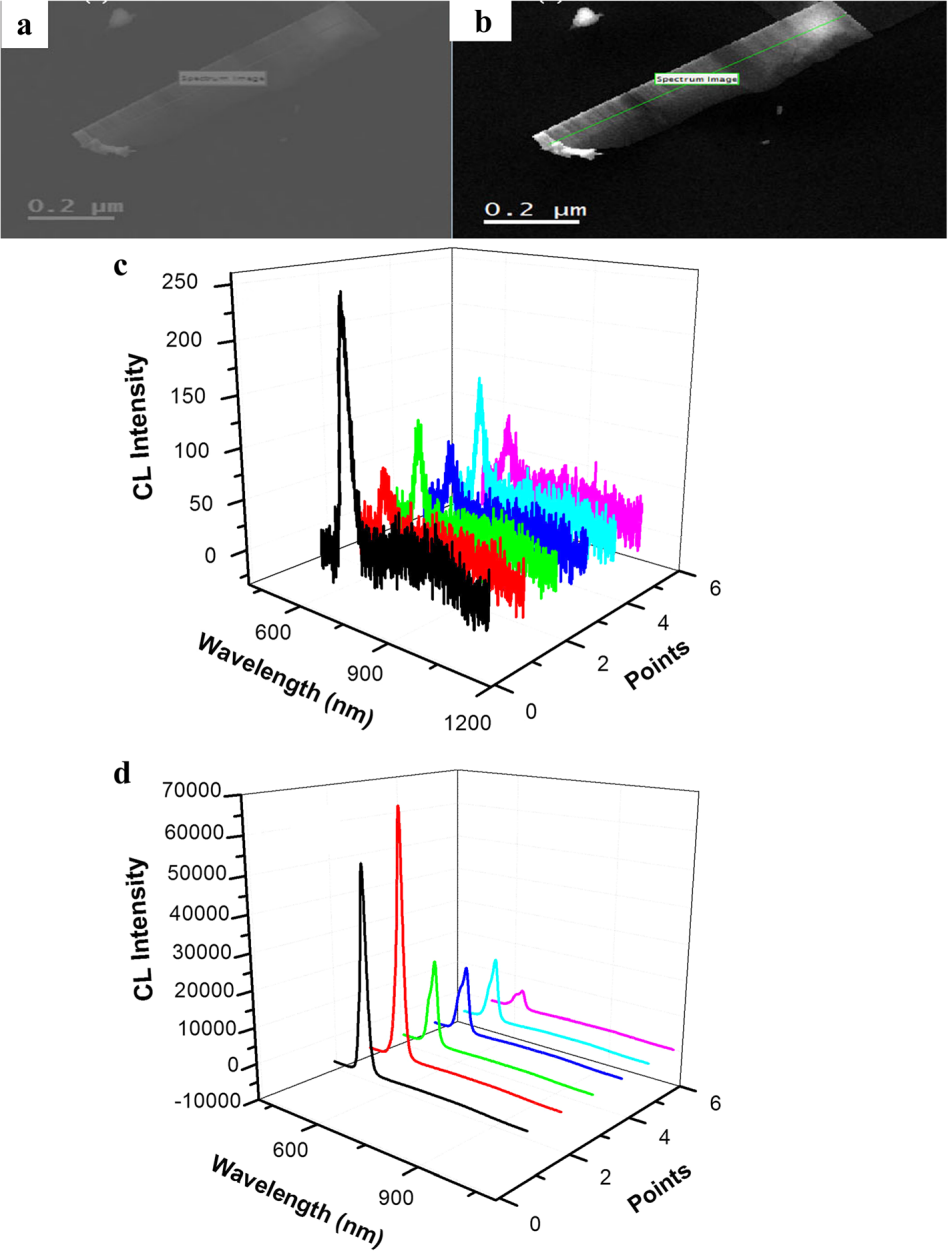
SEM and CL images of a single CdSSe NB. **a** SEM. **b** CL. **c** CL at 295 K. **d** CL at 93 K

SEM and CL images of the single CdSSe nanobelt are described in Fig. 6a, b. It elucidated that the surface of the nanobelts is flat and smooth and the brightness is heterogeneous along its length. Figure 6c, d is the spatial resolution CL spectra of the same nanobelt at room temperature (295 K) and low temperature (93 K). It highlighted that the CL intensities of the characteristic NBE on CdSSe NB are different from point to point, and their signal noise ratio is not good at 295 K, whereas the CLs are strong with different intensities from point to point at 93 K. This result is in good agreement with the CL image. In addition, the characteristic peak is located at 625 nm, no defect emission is observed, and the intensity at 93 K is about 220-fold stronger than that at 295 K. Therefore, the CdSSe NB has good luminescent properties at low temperature.

**Fig. 7:**
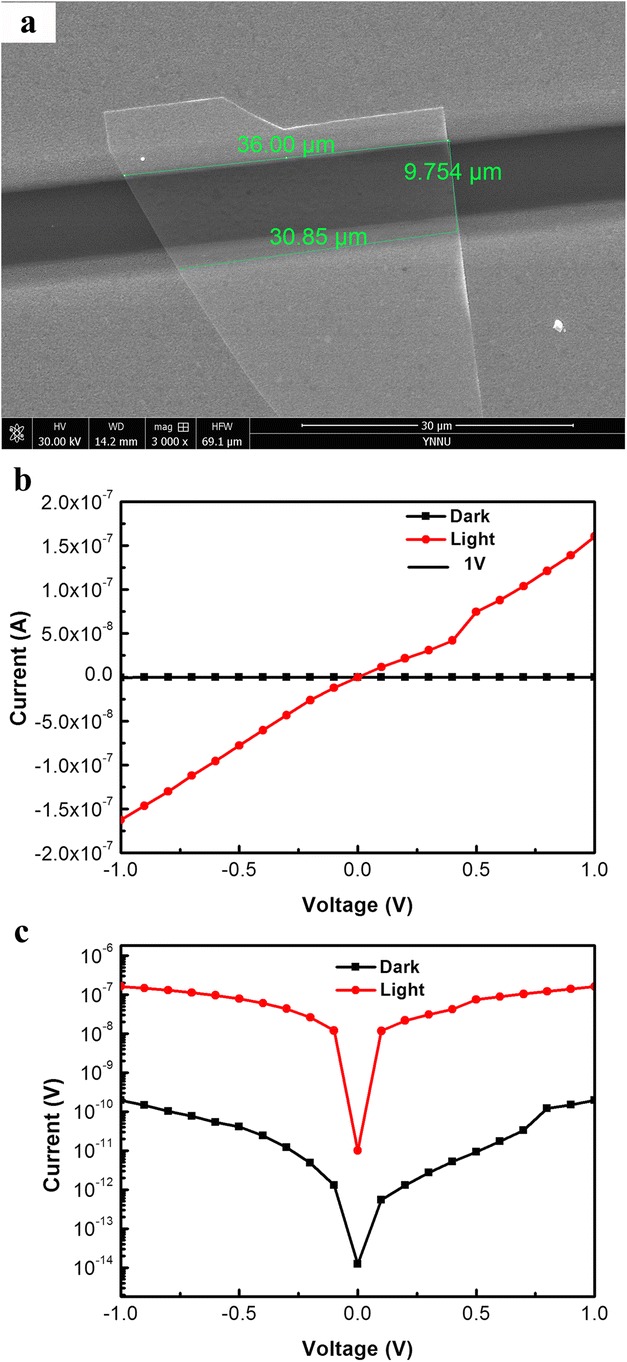
SEM image and *I*–*V* curves of a single CdSSe NB detector. **a** SEM. **b** *I*–*V* curves under dark conditions and white light illumination with power density of 43.14 mW/cm^2^. **c** *I*–*V* graph obtained after taking logarithm

Figure 7a is the SEM image of the nanodevice, which visualized that the CdSSe NB is not uniform in width. The widths of the measured NB are 30.85 and 36 μm and the length is 9.754 μm, as marked in Fig. 7a. The *I*–*V* characteristics of the CdSSe NB device is exhibited in Fig. 7b under dark conditions and white light illumination with power density of 43.14 mW/cm^2^. As can be seen, the photocurrent increases greatly under the white light irradiation, because the incident light produces electron-hole pairs, thus improving the photocurrent. The linear shape of *I*–*V* curve indicated that good ohmic contacts between the CdSSe NB and Ti/Au electrodes were formed. The photocurrent is 1.6 **×** 10^−7^ A, and the dark current is about 1.96 **×** 10^−10^ A. Therefore, the ratio of photocurrent to dark current is 816. Figure 7c is the *I*–*V* curve obtained after taking logarithm and found that the photocurrent is higher by three orders of magnitude than its dark current.

**Fig. 8:**
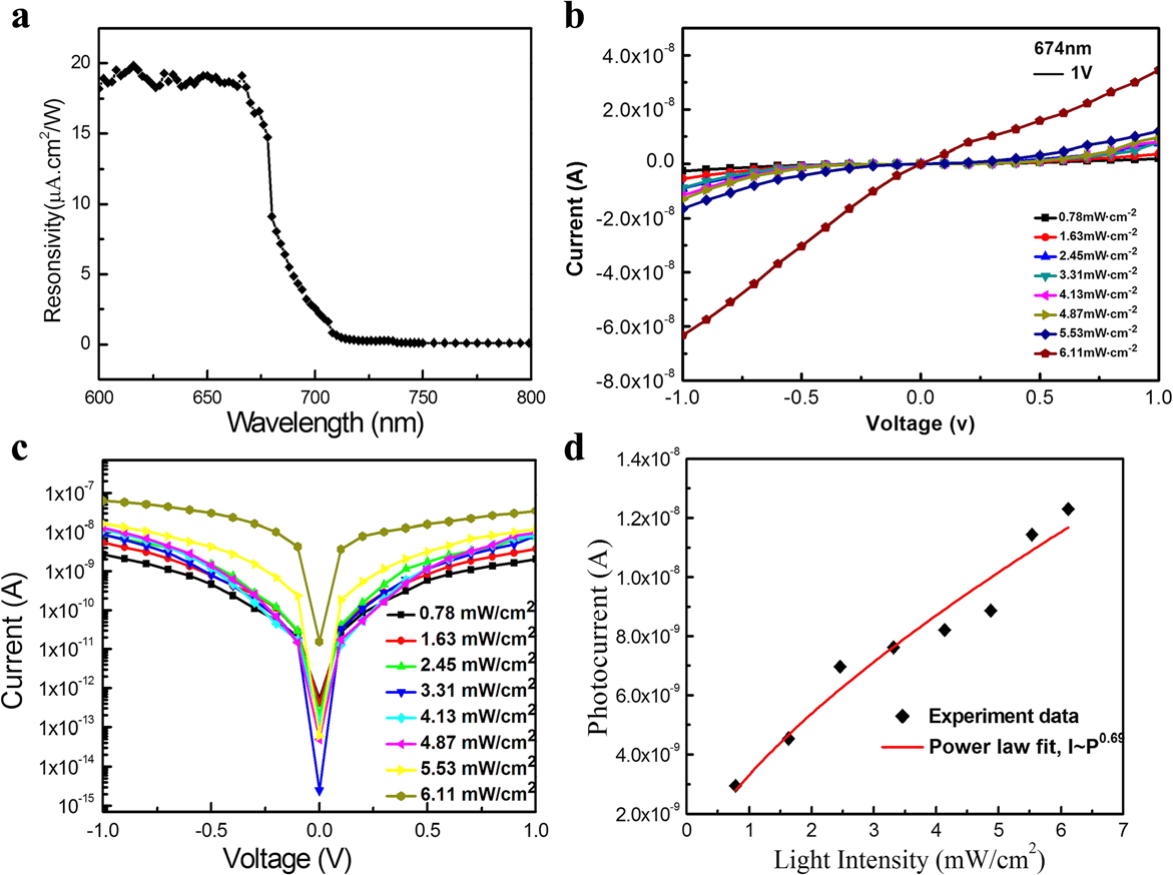
The detector’s photoresponse properties of the CdSSe NB detector. **a** Spectral photoresponse measured at a bias of 1 V. **b** *I*–*V* curve at the excitation wavelength of 674 nm, a bias voltage of 1 V, and different power densities. **c** The logarithmic plot of **b**. **d** The relationship between the photocurrent and the optical power density

To further explore the photoelectronic properties of the devices, we measured the photocurrent of a single CdSSe NB device, as shown in Fig. 8. At an applied biasing voltage of 1 V, the spectral response of the device in the range of 600 to 800 nm is displayed in Fig. 8a. It is seen that the response is very strong as the wavelength is less than 674 nm, and then becomes weaker and weaker when the wavelength is more than 674 nm. Figure 8b presents the measured *I*–*V* curve under illumination of 674 nm light with different power densities. It is found that the photocurrent increases with increasing power density, implying that the photogenerated carrier efficiency is proportional to the number of absorbed photons [30]. The logarithmic plot corresponding to Fig. 8b is highlighted in Fig. 8c. It revealed that the CdSSe NB device has the best response at a power density of 6.11 mW/cm^2^. Figure 8d is the relationship between the photocurrent and the optical power density. By fitting the power density-dependent photocurrent value of *I*_p_ = *AP*^*θ*^, where *I*_p_ is the photocurrent, *P* is the optical power density, *A* is the wavelength-dependent constant, the exponent *θ* determines the photocurrent response with power [31], a good fit of the experimental results has been obtained with *θ* = 0.69. Reports on a non-unity exponent with 0.5 < θ < 1 suggest a complex process of electron–hole generation, recombination, and trapping within the photoactive material [32], whereas the intensity dependence with *θ* < 0.5 may arise owing to defect mechanisms, including both recombination centers and traps. Therefore, *θ* = 0.69 means that CdSSe nanobelt has no defects, which is in agreement with that by HRTEM and CL.

It is well known that the spectral responsivity (*R*_*λ*_) and external quantum efficiency (EQE) are critical parameters for optical devices, which can be defined as *R*_*λ*_ = *I*_ph_/(*P*_*λ*_*S*)and EQE = *hcR*_*λ*_/(*qλ*), where *I*_ph_ is the difference between the photocurrent and dark current, *P*_*λ*_ is the light power density irradiated on the nanobelt, *S* is the effective illuminated area, *c* is the velocity of light, *h* is the Planck’s constant, *q* is the electronic charge, and *λ* is the exciting wavelength [33, 34]. We calculated the corresponding *R*_*λ*_ and EQE values of the CdSSe NB device are 10.4 A/W and 19.1%.

**Fig. 9:**
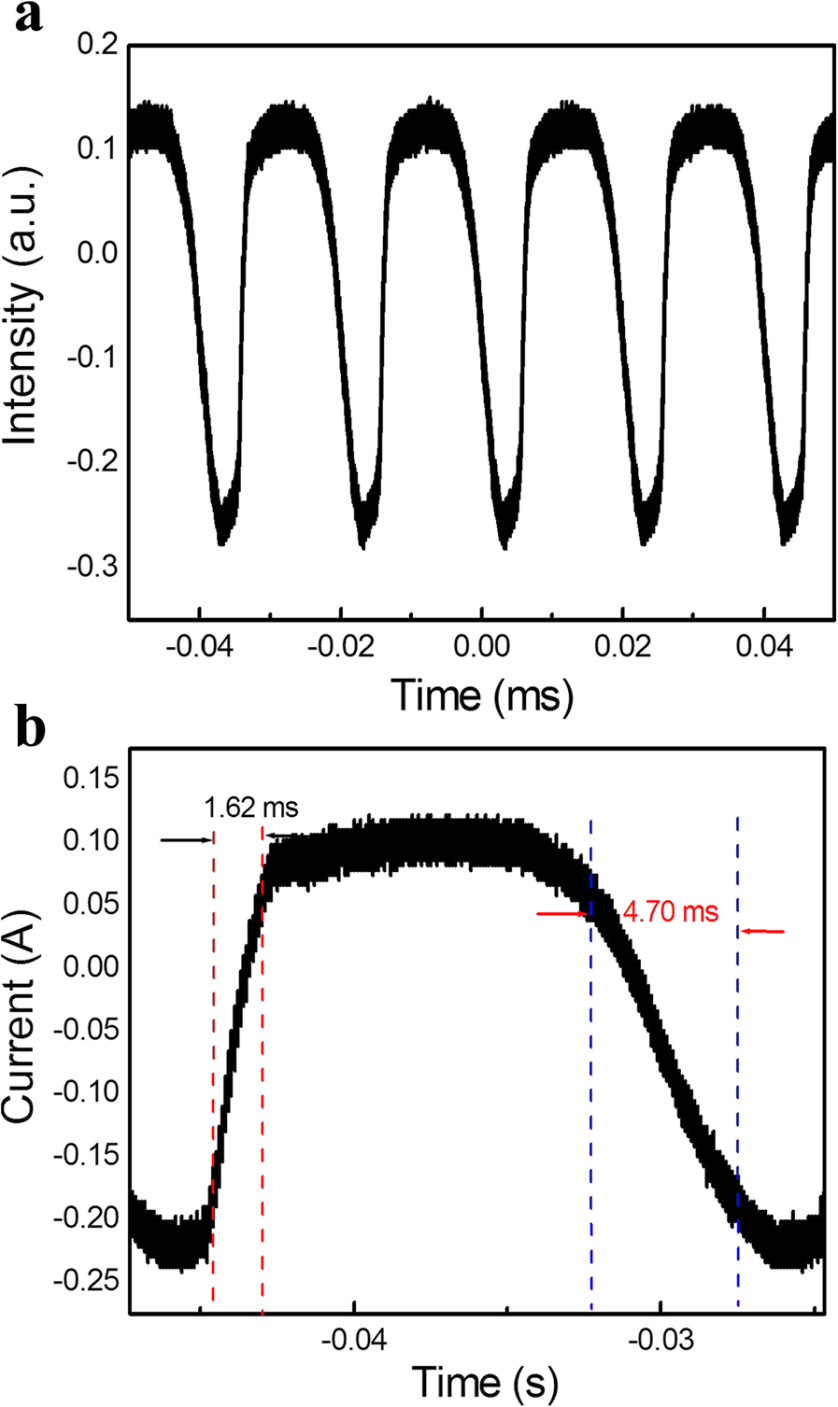
Current–time properties of single CdSSe NB photodetector upon 674-nm light illumination with 4.87 mW/cm^2^ power density under 1 V bias. **a** *I*–*t* characteristics with on/off switching. **b** The voltage rise and decay edge of a pulse response

Figure 9a shows the time response of the CdSSe NB detector, which is measured by periodically turning on and off the 674-nm light with an intensity of 4.87 mW/cm^2^ at a bias voltage of 1 V. From that, we can see that CdSSe NB device exhibits a good reversible stability on switching properties. Figure 9b is the voltage rise and decay edge of a resistant measured by the oscilloscope. It reflects the photoconductance rise time and decay time of CdSSe NB. With and without illumination of 674-nm light (4.87 mW/cm^2^), the voltage of the resistant changes varies. It is seen that the rise/decay time is 1.62/4.70 ms, respectively. We compared important parameters of our photodetector with those of others based on single nanobelt or nanosheet (NS). It is found that the *R*_*λ*_ of the CdSSe NB device in this work is larger than that of other nanostructure photodetectors such as CdS [34] and ZnS NB [35], BiO_2_Se [36], GaSe [37], SnS [38], and Bi_2_S_2_ NS [39]. The decay time is shorter than that of ZnS NB [35] and GaSe NS [37], but longer than that of others [34, 36, 38, 39], as summarized in Table 1, thus confirming the potential application of the CdSSe NB for photodetective field.

**Table 1:** Comparisons of important parameters of photodetectors based on single different nanobelt/nanosheet

Materials	Contact type	Response range	Responsivity	Decay time	Ref
Cds NB	OC	< 500 nm	6	20 μs	[34]
ZnS NB	SC	UV	~ 2	< 0.3 s	[35]
BiO_2_Se NS	OC	808 nm	6.5	2.8 ms	[36]
GaSe NS	SC	800 nm	3.5	0.1 s	[37]
SnS_2_ NS	OC	Visible	8.8 × 10^−3^	5 μs	[38]
Bi_2_S_3_ NS	OC	Visible-NIR	4.4	10 μs	[39]
CdSSe NB	OC	674 nm	10.4	4.7 ms	This work

Abbreviations: *NB* nanobelt, *NS* nanosheet, *OC* ohmic contact, *SC* Schottky contact

## Conclusions

In summary, CdSSe NBs were grown in a high-temperature furnace by thermal evaporation. The obtained nanobelts were characterized by various methods. It is found that the CdSSe NBs have perfect microstructure without any defects and the nanobelts are composed of Cd, Se, and S elements. The CL results revealed that the intensity of single CdSSe nanobelt at low temperature (93 K) is stronger than that at room temperature (295 K), and the signal noise ratio is better at 93 K. After that, we developed the CdSSe photodetector based on single NB and studied its optoelectronic properties. The detector achieved high performance with responsivity of 10.4 A/W, rise/decay time of 1.62/4.70 ms, and the external quantum efficiency (EQE) of 19.1% at 674 nm, which has good stability and repeatability in the photoelectronic properties. This work paves the way towards for developing continuous-wavelength visible photodetector by tuning its composition.

## Abbreviations

CL: Cathodoluminescence
EDX: Energy-dispersive X-ray
EQE: External quantum efficiency
HRTEM: High-resolution electron microscopy
NB: Nanobelt
NBE: Near-band-edge
NS: Nanosheet
PL: Photoluminescence
*R*_*λ*_: Responsivity
SAD: Selected-area diffraction
SEM: Scanning electron microscopy (SEM)
TEM: Transmission electron microscopy
XPS: X-ray photoelectron spectroscopy
XRD: X-ray diffraction
